# Do callers to out-of-hours care misuse an option to jump the phone queue?

**DOI:** 10.1080/02813432.2019.1608067

**Published:** 2019-05-09

**Authors:** J. F. Ebert, L. Huibers, B. Christensen, F. K. Lippert, M. B. Christensen

**Affiliations:** a Research Unit for General Practice, Department of Public Health, Aarhus University, Aarhus C, Denmark;; b Section for General Medical Practice, Department of Public Health, Aarhus University, Aarhus C, Denmark;; c Emergency Medical Services Copenhagen, Capital Region of Denmark, Copenhagen, Denmark

**Keywords:** After-Hours Care, Delivery of Health Care, Health Services Accessibility, Triage, Risk Assessment, General Practice

## Abstract

**Objectives:** Out-of-hours (OOH) services provide access to healthcare outside normal office hours, but the waiting time can sometimes be long. All callers must wait in the telephone queue, even if the health problem is urgent or life-threatening. We tested an emergency access button (EAB), which allowed callers with perceived severe health problems to bypass the queue. We aimed to investigate the severity of the health problems and the relevance of EAB use (assessed by triage professionals). Additionally, we aimed to calculate the number of suspected acute myocardial infarctions (AMI) and ambulance dispatches.

**Design:** Descriptive study of a randomized intervention.

**Setting:** OOH services in two major Danish healthcare regions.

**Subjects:** 217,510 callers participated; 146,355 were randomized to intervention, and 6554 of 6631 (98.8%) questionnaires were completed by OOH triage professionals.

**Intervention:** An EAB allowing randomly selected callers to bypass the telephone queue.

**Main outcome measures:** Severity of contact and relevance of EAB use. Number of suspected AMIs and ambulance dispatches.

**Results:** In both settings, contacts with EAB use concerned significantly more severe health problems than contacts without EAB use (*p* < 0.001). Triage professionals rated EAB use as “not relevant” in 23% of cases. Significantly more EAB users (10.4%) than EAB non-users (3.3% with EAB option and 1.7% without EAB option, *p* < 0.001) had a suspected AMI.

**Conclusions:** We found higher proportions of severe health problems, suspected AMIs, and ambulance dispatches among EAB users. Only 23% of EAB use was rated “not relevant”. This suggests that the EAB is used as intended.

**Key points**
- Out-of-hours healthcare is challenged by increasing demand and long triage waiting times.
- An emergency access button may allow severely ill callers to jump the queue.
- Callers who bypassed the queue were more severely ill than callers who did not bypass the queue.
- Only 23% of bypassers presented “not relevant” health problems according to the triage staff.**Trial registration:** Identifier NCT02572115 registered at Clinicaltrials.gov on 5 October 2015.

## Introduction

Many countries have out-of-hours (OOH) primary care services, emergency departments (ED), and Emergency Medical Dispatch Centers (EMDC-112) [1–3]. Although these target different healthcare needs, they tend to have partly overlapping patient populations.

A Danish study found that approximately 6% of all patients calling a regional OOH primary care service estimated their condition as highly severe or potentially life-threatening, whereas general practitioners (GPs) assessed that 4.5% of callers had a highly severe condition [4]. Studies on urgency levels in the OOH services have shown that 1.3–9.0% of calls are triaged directly to immediate ambulance care [5–8]. Nevertheless, approximately 20% of calls to the EMDC-112 are not relevant for ambulance care [9]. A gray zone thus seems to exist for some patients who may not have contacted the most appropriate service and may suffer because of prolonged waiting time.

The OOH services in Denmark can have telephone waiting times of more than 25 minutes in peak hours (personal communication with staff at the two Danish OOH services explored in this study). Studies have shown that patients with acute myocardial infarction (AMI) who rapidly receive reperfusion therapy have smaller infarct size and lower mortality than patients experiencing treatment delays [10,11]. An option to bypass the telephone waiting line at the OOH service could help ensure that citizens who need immediate medical attention get the right help from the right healthcare professional sooner.

The option to bypass the telephone queue is inspired by a similar option in the Netherlands, but little is known of the extent of use, its effects and the relevance of use [3,12,13]. We have implemented an Emergency Access Button (EAB) at the OOH service in two Danish regions to test its use and feasibility. Approximately 3% of callers chose to use it and bypass the waiting line [14]. In this paper, we aimed to investigate the extent to which this EAB use is perceived as relevant by the triage professionals and to investigate differences in relevance of EAB use and severity of health problems between the two settings. We hypothesize that the reasons for calling are more severe among EAB users than EAB non-users. Furthermore, we aimed to explore if EAB users aged 40+ years were more often suspected of AMI and more often received ambulance care as these two outcomes were considered a proxy for relevant use of the intervention.

## Material and methods

### Design and setting

We conducted a randomized intervention trial at two OOH services using telephone triage: the general practitioner cooperative (GPC) in the Central Denmark Region and the medical helpline 1813 (MH-1813) in the Capital Region of Denmark. These two settings differ on waiting time (GPC mean: 71 sec, ICI: 25%;75%: 11;215 sec; MH-1813 mean: 153 sec, ICI: 25%;75%: 12;420 sec) and on educational background of the triage professionals [12]. In this paper, all employees performing triage at these two call centers are collectively referred to as *triage professionals*.

The OOH service in Denmark is run by GPs organized in large-scale GPCs in four of the five Danish regions [15]. The fifth region has MH-1813, which serves as a publicly run call center using triage by nurses (approx. 80%) and medical doctors with various specialties or in specialty training. The OOH service provides immediate access to healthcare outside normal office hours, i.e. between 4 p.m. and 8 a.m. on weekdays and during all weekends and holidays [15]. Depending on the nature and severity of the presented health problem, different types of care are provided: telephone advice, clinic consultation or home visit (by a doctor), or direct hospital admission by ambulance [14].

All citizens calling the OOH service must wait in queue, regardless of the health problem. The only other option for getting immediate help is the EMDC-112, which is intended for life-threatening situations that require immediate medical response (e.g. ambulance dispatch).

### Intervention

**Figure 1.**
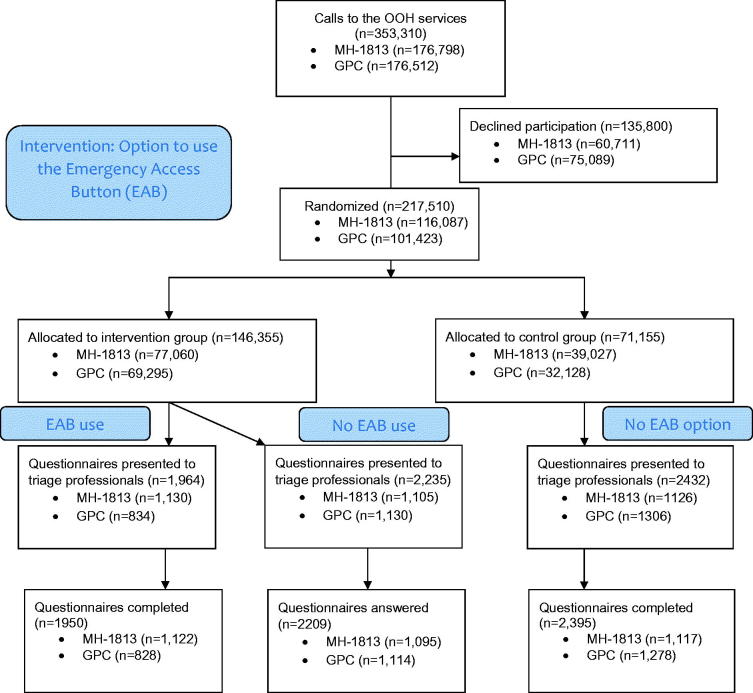
Flow diagram of included subjects (based on CONSORT guidelines). EAB: emergency action button; GPC: general practice cooperative (Central Denmark Region); MH-1813: medical helpline 1813 (Capital Region of Denmark).

All callers were informed of the project and given the opportunity to decline participation through an automated answering service. All callers were also informed of the estimated waiting time. Participants were randomized into two arms based on their date of birth: opportunity to use the EAB option (even date) or regular service (uneven date) (see Figure 1). Callers randomized to the intervention arm could bypass the telephone waiting line by pressing “9”. The message in Danish on the answering machine corresponded to the following: “*If your condition is so severe that you find it necessary to get through straight away, you may press 9 and get first in line. Otherwise please wait”.* Bypassing the telephone waiting line meant jumping to the front of the digital queuing system and becoming next in line to talk to a triage professional.

### Power calculation

The study size was based on the number of completed caller questionnaires needed to ensure sufficient data on satisfaction with the EAB and the contact with the OOH service (not reported here). We needed approximately 300 respondents per group to detect a mean difference of .3 on a 5-point Likert scale, assuming a standard deviation of 1, a significance level of 5%, and a power of 95%. The expected response rate was approx. 35%, and we thus needed to include around 1000 callers per group per setting.

### Data collection

Questionnaire data from triage professionals were collected from 4 September to 30 November 2017 in both settings. We included three study groups: no EAB option, EAB option not used, and EAB option used. During the first eight weeks, all data on the “no EAB option” and “EAB option not used” groups were collected. The data for the” EAB option used” group were collected through the entire study period to obtain the estimated 1000 respondents per group from each setting.

We used pop-up questionnaires on the work stations of the triage professionals. To ensure that the triage professional was “blinded” during the call, the questionnaire did not emerge until the call had been ended. This method has proven feasible for collecting data in earlier studies [4,12,16,17]. In the GPC, the triage professional could decline participation in the study when logging in, whereas participation was mandatory in MH-1813. In both settings, the triage professional could decline completing a questionnaire after having agreed to participate in the study. Non-participating triage professionals could talk to callers who chose to use the EAB, but they did not get a pop-up questionnaire.

### Exclusion and inclusion

All callers and triage professionals were invited to participate in the study period. No questionnaires filled in by triage professionals were excluded from the analyses. Included subjects are shown in Figure 1.

### Questionnaire and definitions

The questionnaire for triage professionals was developed based on previous studies, literature study, and internal feedback rounds using the extensive experience of the author group with OOH service research and clinical triage work. We received external feedback on the wording and the clinical relevance of the questions from five triage professionals in the GPC and MH-1813. We also performed a two-week pilot test at the beginning of 2016 [12], which provided us with feedback from triage professionals who had completed the questionnaire.

The questionnaire contained six questions concerning callers who had used the EAB and five questions concerning callers who had not used the EAB. Four questions (severity of problem, presented symptoms, suggested diagnosis, new or chronic disease) came from an earlier study at the GPC [16]. Two new questions on the relevance of EAB use were developed. These incorporated two types of relevance: strictly medical relevance and relevance seen from an overall perspective. The *medical* necessity of bypassing the queue was assessed on the basis of the presented symptoms. The relevance seen from the *overall perspective* was based on the patient’s overall situation (i.e. symptoms and social or personal motivation). An English version of the questionnaire is available in Appendix 1.

The questions regarding symptoms and probable diagnosis were answered in the pop-up questionnaire by the triage professionals in the GPC, whereas the triage professionals in MH-1813 entered an ICPC-2 code [18] as a standard procedure. We defined “suspected AMI” as a call resulting in ambulance dispatch for a patient older than 40 years for whom the triage professional had answered “suspected AMI” or entered ICPC-2 code K1, K2, K3, or K75 [18]. If a diagnosis was missing, we searched the electronic patient record for entries made by the triage professional stating “heart related chest pain”, “suspected AMI”, or similar unambiguous descriptions of an AMI. All typed-in answers were proofread by a medical doctor (JFE).

### Analyses

We used the Pearson chi-squared test to compare the distribution of sex and age for all three groups, stratified for setting and to analyze if any differences were seen in the distribution of severity and relevance of contacts (measured on a 5-point Likert scale) between the settings and the groups in each setting. We computed a corrected group called “EAB offered (corrected)” that accounted for the fact that we only had information on a random sample (approximately 3% as this was the fraction that used the EAB (14)) from the” EAB offered not used group”. To test for homogeneity in the distribution of severity, suspected AMI and ambulance dispatch between the “EAB offered” and “EAB not offered” groups, we used the Pearson Chi squared test with a Rao Scott second order correction.We used the Pearson Chi squared test to compare the binomial distribution of suspected AMI and ambulance dispatch for the three groups, stratified for setting. Stata 14 was used for the statistical analyses.

## Results

In total, 217,510 of 353,310 (61.6%) callers chose to participate in the study. In total, 6631 participants were randomized to having a questionnaire about their contact triggered to triage professional. In MH-1813, all triage professionals participated. In the GPC, 834 of 1081 (77.1%) EAB users talked to a participating triage professional. A high response rate was seen among participating triage professionals in both settings: 3220/3270 (98.5%) in the GPC and 3334/3361 (99.2%) in MH-1813 (Figure 1).

### Patient characteristics

Table 1 shows the characteristics of patients for whom the triage professionals completed a questionnaire. The distribution of sex in the two settings was similar, but slightly more males were seen among EAB users in MH-1813. Differences between the distribution of the age groups between GPC and MH-1813 were small but significantly different. The actual waiting time was more than twice as long in MH-1813 (153 sec.) as in the GPC (71 sec.) for all callers combined. The estimated waiting time (only available for MH-1813) was three times as long for EAB users (360 sec.) than for EAB non-users (120 sec.).

**Table 1.** Distribution of sex, age group, and waiting time in the study population 2017, *N* = 6554.

Setting	GPC			MH-1813		
Subgroups	EAB not offered	EAB offered not used	EAB used	EAB not offered	EAB offered not used	EAB used
Subgrou	(*n* = 1278)	(*n* = 1114)	(*n* = 828)	(*n* = 1117)	(*n* = 1095)	(*n* = 1122)
**Characteristics**						
Sex (%)						
Male	45.85	44.88	46.98	42.79	43.84	51.25
Female	54.15	55.12	53.02	57.21	56.16	48.75
Age group, years (%)						
0–4	15.41	16.61	9.42	19.07	17.90	15.95
5–13	7.82	8.35	4.95	12.26	12.60	6.60
14–17	3.52	5.30	3.62	5.28	4.66	3.65
18–40	31.77	34.56	27.29	30.08	35.8	26.02
41–60	18.31	18.49	19.69	17.99	15.71	19.88
61–75	12.83	8.98	19.08	9.04	8.49	14.53
≥76	10.33	7.72	15.94	6.27	4.84	13.37
Waiting time, seconds						
Actual, median	75	73	25	137	173	46
IQI: p25-p75	12;223	11;217	11;59	10;390	12;442	34;66
Estimated, median	–	–	–	120	120	360
IQI: p25-p75	–	–	–	0;300	0;360	180;660

No significant differences were seen in the distribution of sex in the GPC for the three groups. In MH-1813, the distribution of sex in the “EAB used” subgroup was significantly different from the other two groups (*p* < 0.001) when using the chi-squared test.

The distribution of age groups was significantly different between all groups and settings when comparing individually using the chi-squared test, except between the “EAB not offered” and “EAB offered not used” groups in the MH-1813 (*p* = 0.094).

EAB: emergency action button; GPC: general practice cooperative (Central Denmark Region); MH-1813: medical helpline 1813 (Capital Region of Denmark); IQI: interquartile range.

### Severity

We found no significant differences in the severity between the groups that were not offered the EAB and the groups that were offered the EAB but chose not to use it regarding children in both settings and adults in MH-1813. However, the health problems in adults contacting the GPC without getting the EAB option were assessed as significantly more severe than the health problems in the group who did get the EAB option but chose not to use it (*p* = 0.036) (Table 2). Callers who used the EAB were assessed to have considerable and significantly more severe health problems than the group who did not use the EAB and the group who did not get the option (combined: severe, potentially life-threatening adults EAB used: 18.5% vs. EAB not offered: 5.2% and EAB offered but not used: 4.8%; chi-squared test for distribution: *p* < 0.001).

**Table 2.** Severity of contacts for adults and children aged <18 years as assessed by triage professionals 2017, *N* = 6554 (%).

		EAB not offered		EAB offered not used		EAB used		EAB offered (corrected)	
Setting	Group	Adult	Child	Adult	Child	Adult	Child	Adult	Child
GPC		*n* = 936	*n* = 342	*n* = 777	*n* = 337	*n* = 679	*n* = 149		
	Severe, potentially life threatening	3.7	*n* < 5	3.3	*n* < 5	17.7	9.4	3.7	0.7
	Severe, not life threatening	17.3	12.0	13.9	11.3	26.1	30.9	14.2	11.5
	Not severe but illness	56.6	68.4	59.2	65.3	44.0	50.3	58.8	65.2
	Not severe	19.1	15.8	22.0	20.8	9.1	8.1	21.7	20.5
	Don't know	3.2	3.5	1.5	2.1	3.1	*n* < 5	1.6	2.1
MH-1813		*n* = 708	*n* = 409	*n* = 710	*n* = 385	*n* = 828	*n* = 294		
	Severe, potentially life threatening	7.1	3.4	6.5	2.6	19.3	6.1	6.9	2.7
	Severe, not life threatening	17.8	12.2	14.5	10.9	24.5	18.0	14.8	11.1
	Not severe but illness	28.1	37.7	35.1	36.9	25.0	34.0	34.8	36.8
	Not severe	42.4	44.3	39.9	47.0	27.3	38.8	39.5	46.8
	Don't know	4.7	2.4	4.1	2.6	3.9	3.1	4.1	2.6
Combined		*n* = 1644	*n* = 751	*n* = 1487	*n* = 722	*n* = 1507	*n* = 443		
	Severe, potentially life threatening	5.2	*n* < 19	4.8	*n* < 15	18.5	7.2	5.2	1.8
	Severe, not life threatening	17.5	12.1	14.2	11.1	25.1	22.3	14.5	11.3
	Not severe but illness	44.3	51.7	47.7	50.1	33.5	39.5	47.3	50.0
	Not severe	29.1	31.3	30.5	34.8	19.4	28.4	30.2	34.6
	Don't know	3.8	2.9	2.8	2.4	3.5	*n* < 14	2.8	2.4

Results with less than five cases are not reported due to data protection regulations by the Danish Data Protection Agency. These cases are instead labelled “*n* < 5”. In the “combined” section, this is also the case as a percentage would give away results labelled *n* < 5.

EAB not offered vs. EAB offered not used: *Adults*: GPC *p* = 0.036; MH-1813: *p* = 0.066; Combined: *p* = 0.030. *Children*: GPC *p* = 0.377; MH-1813: *p* = 0.890; Combined: *p* = 0.637. Analysed using the Pearson chi squared test for differences in distribution of answers.

EAB not offered vs. EAB offered (corrected): *Adults*: GPC *p* = 0.036; MH-1813: *p* = 0.099; Combined: *p* = 0.0501. *Children*: GPC *p* = 0.348; MH-1813: *p* = 0.910; Combined: *p* = 0.699. Analysed using the using the Pearson chi squared test with a Rao Scott second order correction. To account for the fact that we only had information on a random sample (approximately 3%) from the” EAB offered not used”-group we applied a sampling weight of approximately 33 in that group when we calculated the test-statistic.

All distributions in the “EAB used” group are significantly different from the “EAB offered not used” and “EAB not offered” groups (*p* < 0.001), except from the “EAB not offered” group for children in MH-1813 (*p* = 0.067).

EAB: emergency action button; GPC: general practice cooperative (Central Denmark Region); MH-1813: medical helpline 1813 (Capital Region of Denmark).

### Relevance

The triage professionals generally found EAB use to be more relevant for calls concerning adults than for calls concerning children. This trend was most profound for assessments based on medical relevance, but a similar trend was also seen for assessments based on the overall perspective. In both settings, calls regarding children were more often perceived as “not relevant” than calls regarding adults. In MH-1813, the proportion of “not relevant” EAB use was significantly higher than in the GPC (medical relevance: 50.2% vs. 35.6%, *p* = 0.0035; overall perspective 33.0% vs. 22.8%; *p* = 0.0262) (Table 3).

**Table 3.** Relevance of choice to use the EAB for adults and children aged <18 years as assessed by triage professionals 2017, *N* = 1950 (%).

		GPC		MH-1813		Combined			
	Setting	Adults	Children	Adults	Children	Adults	Children	P-value	
		*n* = 679	*n* = 149	*n* = 828	*n* = 294	*n* = 1507	*n* = 443	Adults	Children
Strictly medical (%)								0.01	0.05
	Very relevant	19.4	12.8	20.3	9.2	19.9	10.4		
	Relevant	17.4	18.1	20.3	15.0	19.0	16.1		
	Less relevant	27.2	32.9	20.8	24.2	23.7	27.1		
	Not relevant	34.5	35.6	35.3	50.2	34.9	45.2		
	Don't know	1.5	<5 (.)	3.3	<5 (.)	2.5	<10 (.)		
Overall perspective (%)								0.00	0.02	
	Very relevant	23.9	18.8	26.7	14.4	25.4	15.9			
	Relevant	34.3	36.9	27.1	26.1	30.3	29.8			
	Less relevant	21.8	20.8	16.9	22.7	19.1	22.0			
	Not relevant	17.7	22.8	24.8	33.0	21.6	29.5			
	Don't know	2.4	<5 (.)	4.6	3.8	3.6	<16 (.)			

Results with less than five cases are not reported due to data protection regulations by the Danish Data Protection Agency. These cases are instead labelled “*n* < 5”. P-values in the table refer to the chi-square test for the same age group (*adults GPC vs. adults MH-1813, children GPC vs. children MH-1813*) in each setting.

Adults are compared to children within settings with chi-square test for *medical relevance*: adults vs children at GPC: *p* = 0.283, adults vs children at MH-1813: *p* < 0.001. Adults are compared to children in both settings with chi-square test for relevance seen from an *overall perspective*: adults vs children at GPC *p* = 0.278, adults vs children at MH18-13 *p* < 0.001.

EAB: emergency action button; GPC: general practice cooperative (Central Denmark Region); MH-1813: medical helpline 1813 (Capital Region of Denmark).

### AMI and ambulance dispatch

Table 4 describes the number of suspected AMIs and ambulance dispatches. In the GPC, we identified 47/453 (10.4%) suspected AMIs in the group with EAB use, which was significantly more than in the group with EAB option and no use (3.3%) and the group without EAB option (1.7%) (*p* < 0.001). There was no significant trend regarding AMIs in MH-1813. Significantly more ambulances were dispatched in both settings to patients with EAB use compared to the other groups, which means that an EAB user was more likely to receive ambulance care compared to EAB non-users.

**Table 4.** Number of suspected AMI in patients aged >40 years and total ambulance dispatches in the GPC and MH-1813 based on questionnaire responses from triage professionals 2017, *N* = 6554.

	No option	EAB option but not use	EAB use	EAB offered (corrected)^b^	Total	*p* Value^a^	Missings / *N* (Pct)
**OOH-PC**							
AMI >40 years, *n* (%)	530 (38.5)	392 (28.5)	453 (32.9)		1375 (100.0)		
No, *n* (%)	521 (98.3)	379 (96.7)	406 (89.6)	(96.4)	1306 (95.0)		
Yes, *n* (%)	9 (1.7)	13 (3.3)	47 (10.4)	(3.6)	69 (5.0)	0.00	0 / 1375 (0.00)
Ambulance, *n*	1278 (39.7)	1114 (34.6)	828 (25.7)		3220 (100)		
No, *n* (%)	1243 (97.3)	1091 (97.9)	726 (87.7)	(97.7)	3060 (95.0)		
Yes, *n* (%)	35 (2.7)	23 (2.1)	102 (12.3)	(2.3)	160 (5.0)	0.00	0 / 3220 (0.00)
**MH-1813**							
AMI >40 years, *n* (%)	372 (30.3)	318 (25.9)	536 (43.7)		1226 (100.0)		
No, *n* (%)	345 (95.3)	290 (93.9)	472 (93.3)	(93.8)	1107 (94.1)		
Yes, *n* (%)	17 (4.7)	19 (6.1)	34 (6.7)	(6.2)	70 (5.9)	0.46	49 / 1226 (4.00)
Ambulance, n (%))	1117 (33.5)	1095 (32.8)	1122 (33.7)		3334 (100.0)		
No, *n* (%)	1070 (95.8)	1045 (95.4)	961 (85.7)	(95.2)	3076 (92.3)		
Yes, *n* (%)	47 (4.2)	50 (4.6)	161 (14.3)	(4.8)	258 (7.7)	0.00	0 / 3334 (0.00)

Table lists total number of suspected AMIs in patients over 40 years where an ambulance was dispatched and total number of ambulances dispatched in each group. Number of suspected AMIs is based upon questionnaire information from triage professionals.

^a^The Chi Squared test test was used.

^b^The test for homogeneity between the intervention groups “EAB offered (corrected)” and “EAB not offered”, were calculated using the Pearson Chi Squared test with a Rao Scott second order correction. To account for the fact that we only had information on a random sample (approximately 3%) from the” EAB offered not used” group we applied a sampling weight of approximately 33 in that group when we calculated the test-statistic.

AMI: acute myorcardial infarction; EAB: emergency action button; GPC: general practice cooperative (Central Denmark Region); MH-1813: medical helpline 1813 (Capital Region of Denmark).

## Discussion

### Principal findings

The level of perceived severity of the health problem was higher among EAB users than among EAB non-users. The triage professionals generally assessed the relevance of EAB use to be higher from an overall perspective than from a strictly medical perspective. Approx. 22–30% of EAB use was assessed as not relevant. In the GPC, the triage professionals suspected AMI in 10.4% of the contacts involving EAB use, which was significantly higher than in contacts without EAB use and contacts without the EAB option. Furthermore, in both settings, significantly more ambulances were dispatched for EAB users.

### Strengths and limitations

Strengths of this study are the low risk of recall bias because the pop-up questionnaire was answered immediately after the contact and the high response rate among participating triage professionals (>98%). However, 22.9% of EAB users in the GPC talked to a triage professional who did not participate in the study, which resulted in missing questionnaire data and potential selection bias. Still, there is no indication that these callers systematically differ from the included callers because non-participation among triage professionals was distributed evenly in the study period. However, we cannot rule out that the non-participating GPs differ from the participating GPs in their assessment of severity and relevance.

An additional factor that might have caused selection bias is that approximately 38.5% of callers declined participation. We have no background information on these patients, but our data (not presented) showed that proportionally more callers declined participation when calling within the first two opening hours (4–6 pm) on Monday to Friday when reasons for calling tend to be less serious [17]. However, some callers might have declined participation because of distress from a severe condition. Thus, non-participation could bias the results in both directions. Also, the internal validity of the study is strengthened, as the weighted group “EAB offered (corrected)” is not statistically significantly different from the “EAB not offered” group, meaning that selection bias in the randomized study groups is less likely.

The triage professionals had different professional backgrounds in the two settings; GPs or doctors in their final year of GP training in the GPC and nurses or doctors of different medical specialties in MH-1813. These different backgrounds and levels of experience could have influenced their assessment of the level of severity and relevance, which might have led to the differences found between the two settings. Moreover, relevance seen from an overall perspective is a more subjective measure that encompasses the evaluator’s empathy. However, it is difficult to declare a consistent difference between the staff at the two settings based solely on their profession.

Another source for potential bias could be that the triage professionals, when filling in the pop-up questionnaire, were given the information that the caller had either used or not used the EAB; this could have affected the triage professionals’ assessment of relevance and severity. However, assessment bias could have gone in either direction.

The difference in the registration of a probable diagnosis (GPC: free-text format; MH-1813: registration of ICPC-2 code) may have resulted in registration of more symptoms than an actual diagnosis in MH-1813, which could have made it more difficult to identify documentation of a suspected AMI. Thus, underestimation of suspected AMI cannot be ruled out in MH-1813.

### Findings in relation to other studies

Nørøxe et al. found that 23.7% of calls to the GPC in the Central Denmark Region were perceived as medically inappropriate by the triage professional [17]. This number aligns with the level of severity in calls to the GPC regarding adults who did not use the EAB (19.1–22.0% were assessed as “not severe”) although a direct parallel cannot be drawn between the two terms. The distribution of the assessed severity of contacts to the GPC shows a similar trend as that found in a study from 2012 exploring the reasons for encounter in OOH primary care in the same region in Denmark [4]. However, far more calls were assessed as “not severe” in MH-1813; this was seen for both EAB users and non-users and for both adults and children (range: 27.3%; 47.0%). When pooled with the response option “ill, but not severely” (range: 52.3%; 83.9%), this proportion is comparable to the level of non-urgent calls in the recently launched NHS 111 in the UK, where an average of 79.0% of calls were classified as “non-urgent” [19].

A clear trend in both settings was the percentage of “not severe” contacts and “not relevant” use of the EAB in calls regarding children aged <18 years; this was especially evident at MH-1813. The relatively higher percentage of non-severe/irrelevant contacts for children than for adults has also been reported in other studies on OOH services and emergency departments [2,17,20,21].

In the GPC, the triage professionals suspected AMI in 13/392 (3.3%) callers aged 40+ years who were offered the EAB option without using it. Moth et al. found that AMI was suspected in 2.5% of individuals aged 51+ years [4]. However, among callers aged 40+ years who used the EAB, 10.4% had a suspected AMI. A Danish study from 2015 on contact patterns in the EMDC-112 found that 11% of contacts were due to “chest pain/heart disease” [22]. Pope et al. found that 11.3% of eligible calls to the NHS 111 resulted in emergency ambulance dispatch [19]. This resembles the number of ambulance dispatches that we found for EAB users (GPC: 12.3%; MH-1813: 14.3%).

## Conclusion

This study shows that the EAB is mostly used in a relevant way. This finding is based on the evaluations made by the triage professionals and on the higher numbers of ambulance dispatches and AMIs among callers who used the EAB.

## Implications for future practice

The EAB can provide faster access to health advice for those in need of immediate care. The option to bypass the queue can help service providers ensure that the patients most in need get help quickly, also during peak hours with long waiting time. Approximately 3% used the EAB [14], and less than 1% of callers are thus expected to misuse the EAB option in the general population. We believe that our results justifies a recommendation of the EAB to be used in the Danish OOH services. Furthermore, we feel that our results could be generalized to other countries. Future research could explore the pathways of EAB users in terms of subsequent contacts to the healthcare system, future medical history, and mortality rates.

This project prompted MH-1813 to implement the EAB in the daily services from November 2018.

## Data Availability

The EU regulations on the protection of personal data implies that the authors are not authorised to share non-aggregated data with a third party. However, data are available from Jonas F Ebert on reasonable request.

## References

[1] Huibers L, Giesen P, Wensing M, et al. Out-of-hours care in western countries: assessment of different organizational models. BMC Health Serv Res. 2009;9:105. 19549325 10.1186/1472-6963-9-105 PMC2717955

[2] Keizer E, Smits M, Peters Y, et al. Contacts with out-of-hours primary care for nonurgent problems: patients' beliefs or deficiencies in healthcare? BMC Fam Pract. 2015;16:157. 26510620 10.1186/s12875-015-0376-9 PMC4625560

[3] Grol R, Giesen P, van Uden C After-hours care in the United Kingdom, Denmark, and the Netherlands: new models. Health Aff (Millwood). 2006;25:1733–1737. 17102200 10.1377/hlthaff.25.6.1733

[4] Moth G, Flarup L, Christensen MB, et al. Kontakt- og sygdomsmønsteret i laegevagten LV-KOS 2011 [Survey on reasons for encounters and disease patterns in OOH primary care LV-KOS 2011] Available from: http://feap.au.dk/fileadmin/feap/LV-KOS_2011_Rapport.pdf.

[5] Moth G, Huibers L, Christensen MB, et al. Out-of-hours primary care: a population-based study of the diagnostic scope of telephone contacts. Famprj. 2016;33:504–509. 10.1093/fampra/cmw048 27328678

[6] Philips H, Van Bergen J, Huibers L, et al. Agreement on urgency assessment between secretaries and general practitioners: an observational study in out-of-hours general practice service in Belgium. Acta Clin Belg. 2015;70:309–314. 25819448 10.1179/2295333715Y.0000000017

[7] Zakariassen E, Hansen EH, Hunskaar S Incidence of emergency contacts (red responses) to Norwegian emergency primary healthcare services in 2007–a prospective observational study. Scand J Trauma Resusc Emerg Med. 2009;17:17–30. 19586530 10.1186/1757-7241-17-30 PMC2725029

[8] Anderson A, Roland M Potential for advice from doctors to reduce the number of patients referred to emergency departments by NHS 111 call handlers: observational study. BMJ Open 2015;5:009444. 10.1136/bmjopen-2015-009444 PMC4663401 26614624

[9] Lehm KK, Andersen MS, Riddervold IS Non-urgent emergency callers: characteristics and prognosis. Prehosp Emerg Care. 2017;21:166–173. 27629892 10.1080/10903127.2016.1218981

[10] Cannon CP, Gibson CM, Lambrew CT, et al. Relationship of symptom-onset-to-balloon time and door-to-balloon time with mortality in patients undergoing angioplasty for acute myocardial infarction. JAMA 2000;283:2941–2947. 10865271 10.1001/jama.283.22.2941

[11] Reed GW, Rossi JE, Cannon CP Acute myocardial infarction. Lancet. 2017;389:197–210. 27502078 10.1016/S0140-6736(16)30677-8

[12] Ebert JF, Huibers L, Lippert FK, et al. Development and evaluation of an "emergency access button" in Danish out-of-hours primary care: a study protocol of a randomized controlled trial. BMC Health Serv Res 2017;17:379. 28566087 10.1186/s12913-017-2308-y PMC5452428

[13] Giesen P, Smits M, Huibers L, et al. Quality of after-hours primary care in the Netherlands: a narrative review. Ann Intern Med. 2011;155:108–113. 21768584 10.7326/0003-4819-155-2-201107190-00006

[14] Ebert JF, Huibers L, Christensen B, et al. Giving callers the option to bypass the telephone waiting line in out-of-hours primary services: a comparative intervention study Submitted to Scand J Prim Health Care 2019 Mar;37:120–127. 10.1080/02813432.2019.1569427 PMC6452808 30712448

[15] Christensen MB, Olesen F Out of hours service in Denmark: evaluation five years after reform. BMJ. 1998;316:1502–1505. 9582141 10.1136/bmj.316.7143.1502 PMC28553

[16] Flarup L, Moth G, Christensen MB, et al. A feasible method to study the Danish out-of-hours primary care service. Dan Med J. 2014;61:A4847 24814746

[17] Noroxe KB, Huibers L, Moth G, et al. Medical appropriateness of adult calls to Danish out-of-hours primary care: a questionnaire-based survey. BMC Fam Pract 2017;18:34. 28292257 10.1186/s12875-017-0617-1 PMC5351208

[18] WHO ICPC-2 2018 [cited 2018 Apr 5]. Available from: http://www.who.int/classifications/icd/adaptations/icpc2/en/

[19] Pope C, Turnbull J, Jones J, et al. Has the NHS 111 urgent care telephone service been a success? Case study and secondary data analysis in England. BMJ Open. 2017;7:e014815 10.1136/bmjopen-2016-014815 PMC5623427 28576895

[20] McHale P, Wood S, Hughes K, et al. Who uses emergency departments inappropriately and when - a national cross-sectional study using a monitoring data system. BMC Med 2013;11:11–258. 24330758 10.1186/1741-7015-11-258 PMC3886196

[21] Carret ML, Fassa AC, Domingues MR Inappropriate use of emergency services: a systematic review of prevalence and associated factors. Cad Saude Publica. 2009;25:7–28. 19180283 10.1590/s0102-311x2009000100002

[22] Moller TP, Ersboll AK, Tolstrup JS, et al. Why and when citizens call for emergency help: an observational study of 211,193 medical emergency calls. Scand J Trauma Resusc Emerg Med. 2015;23:88. 26530307 10.1186/s13049-015-0169-0 PMC4632270

